# Ni-Mn-Sn-Cu Alloys after Thermal Cycling: Thermal and Magnetic Response

**DOI:** 10.3390/ma14226851

**Published:** 2021-11-13

**Authors:** Asma Wederni, Mihail Ipatov, Julián-María González, Mohamed Khitouni, Joan-Josep Suñol

**Affiliations:** 1 Department of Physics, P2, EPS, University of Girona, 17003 Girona, Spain; asma.wederni@gmail.com; 2 Department of Applied Physics, University of the Basque Country, 20018 Donostia, Spain; mihail.ipatov@ehu.es (M.I.); julianmaria.gonzalez@ehu.es (J.-M.G.); 3 Laboratory of Inorganic Chemistry, University of Sfax, B.P. 1171, Sfax 3000, Tunisia; mohamed.khitouni@fss.rnu.tn

**Keywords:** Heusler, thermal cycling, thermal analysis, magnetic behavior

## Abstract

Heusler Ni-Mn-Sn-based alloys are good candidates for magnetic refrigeration. This application is based on cycling processes. In this work, thermal cycles (100) have been performed in three ribbons produced by melt-spinning to check the thermal stability and the magnetic response. After cycling, the temperatures were slowly shifted and the thermodynamic properties were reduced, the entropy changed at about 3–5%. Likewise, the thermomagnetic response remains similar. Thus, these candidates maintain enough thermal stability and magnetic response after cycling. Likewise, Cu addition shifts the structural transformation to higher temperatures, whereas the Curie temperature is always near 310 K. Regarding magnetic shape memory applications, the best candidate is the Ni_49_Mn_36_ Sn_14_Cu_1_ alloy.

## 1. Introduction

Ni-Mn-Sn-based Heusler ferromagnetic alloys are multifunctional materials due to the coupling of a reversible solid-state first order transformation (austenite to martensite) and a second order magnetic transformation (ferromagnetic to paramagnetic). In these alloys, the transformations can be induced by mechanical stress, temperature and/or external magnetic field. Functional effects of these materials are: magnetic shape memory [1], magnetocaloric [2], magneto-resistance [3], exchange-bias [4], barocaloric [5] or elastocaloric [6].

Addition of small quantities of a fourth element in the ternary off-stoichiometry Heusler alloys has been proposed as a way to improve the functional response and to tailor the transition temperatures [7]. The shift of the transition temperatures is due to the sensitivity to interatomic distances and hybridization [8]. This trend opens the possibility of deliberate changes in the transition temperatures and benefits while forcing phase transitions by a temperature and magnetic field [9]. The improvement of the magnetic response could be favored by the addition of a magnetic element as Fe [10] or Co [11]. Gd-based and Ni-Mn-In alloys are good candidates for magnetic refrigeration devices. Thus, Gd [12] or In [13] addition has been also analyzed. A minor amount of other metallic elements, such as Zn [14], Al [15] or Pd [16], or metalloids, such as B [17] have been also checked. In these compounds, the main effect is the shift of the transition temperatures. Obviously, the addition of two elements to combine their effect has been done, such as Pt and Co [18]. In our work, Cu addition has been performed.

Likewise, for the magnetic refrigeration or shape memory applications it is necessary to check the stability of the functional response after cycling. The mechanical cycling from Ni-Mn-based alloys shows that the mechanical stability is linked to the good geometrical compatibility at the interphase between austenite and martensite [19].

In our study, we produce three Ni-Mn-Sn-(Cu) Heusler alloys. In a previous work [20], we analyzed the microstructure of the as produced ribbons as well as the thermal and magnetic behavior. These ribbon flakes have been now subjected to controlled thermal cycling (100 cycles). The main objective is to analyze the thermal and magnetic response to check its thermal and magnetic stability after cycling.

## 2. Materials and Methods

Three alloys with nominal composition (at.%) Ni_50−*x*_Mn_36_Sn_14_Cu_x_ (*x* = 0, 1, and 2) were produced by melt-spinning (melt-spinner MSP 10, Edmund Bühler GmbH, Bodelshausen, Germany) as ribbon flakes (1 mm thick, 2–3 cm long, 20 μm width). The X-ray diffraction (XRD, D500 S equipment, Bruker, Billerica, MA, USA) patterns at room temperature were made to verify the crystallographic structure of the spun-ribbons. The refinement of crystalline structures were made by applying the Maud software, which is founded on the Rietveld method [11]. Austenite to martensite direct and inverse temperatures were determined by differential scanning calorimetry (DSC) in a DSC822 Mettler-Toledo calorimeter, at a heating/cooling rate of 10 K/min. Thermal cycling (100 cycles) experiments were performed with liquid nitrogen between nitrogen liquidus temperature and room temperature. Thermomagnetic measurements were done on a PPMS 6000 device (Quantum Design, San Diego, CA, USA). Zero-field-cooling (ZFC), field-cooling (FC) and field-heating (FH) protocols, in a temperature range from low temperature up to 400 K and an applied magnetic field up to 50 kOe; whereas magnetic hysteresis loops were recorded at 50 K and 300 K.

## 3. Results and Discussion

The results include the crystallographic analysis of the as-quenched ribbon flakes and the analysis of the thermal and magnetic behavior after cycling. These results were discussed by comparison with those of ribbons before thermal cycling.

### 3.1. Crystallographic Analysis

**Figure 1:**
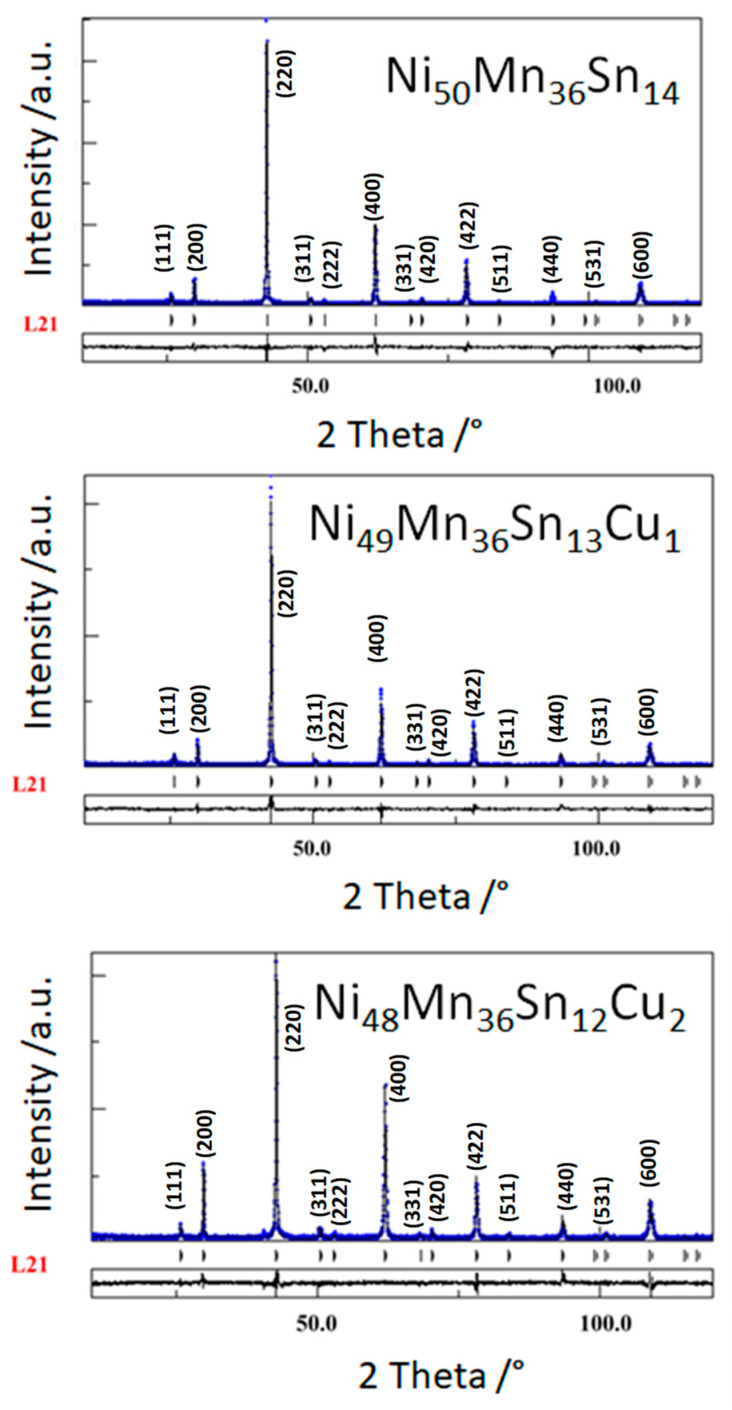
X-ray diffraction patterns of the Ni-Mn-Sn-(Cu) ribbons.

The analysis of the three diffraction patters collected by XRD of the ribbons shows that at room temperature all the samples have a cubic L2_1_ Heusler structure. This crystallographic structure is found in ternary alloys and the stoichiometry is 2:1:1 (X_2_YZ). Thus, we produce of stoichiometry alloys, and the excess od Mn atoms will be in Z sites. The Rietveld analysis has been performed, as shown in the Figure 1.

The L2_1_ orders have been determined by taking into account Equation (1):

(I_111_∕I_220_) _exp_ = [ S_L21_ (3 − S_B2_∕2)]^2^ (I_111_∕I_220_) _th_, (1)

where the I (hkl) refers to the Bragg peak’s intensity, Miller indices (hkl) and the suffixes ‘exp’ represent experimentally obtained intensity values and “th” represents theoretically simulated and “S” represents the order parameter. This order parameter before cycling ranged between 85–88% [20], whereas between 73–75% after cycling. The produced alloys were off-stoichiometry. Thus, this reduction indicates that the alloys evolve to the lower expected L2_1_ order.

The crystallographic parameters derived from Rietveld analysis of the specimens before and after thermal cycling are given in Table 1.

The lattice parameters change slightly with the thermal cycling and/or the Cu addition. Meanwhile, the microstrain decreases after cycling and the crystalline size increases. Probably the local atomic displacements during cycling and the accommodation of the martensite favors this behavior.

### 3.2. Thermal Analysis

**Figure 2:**
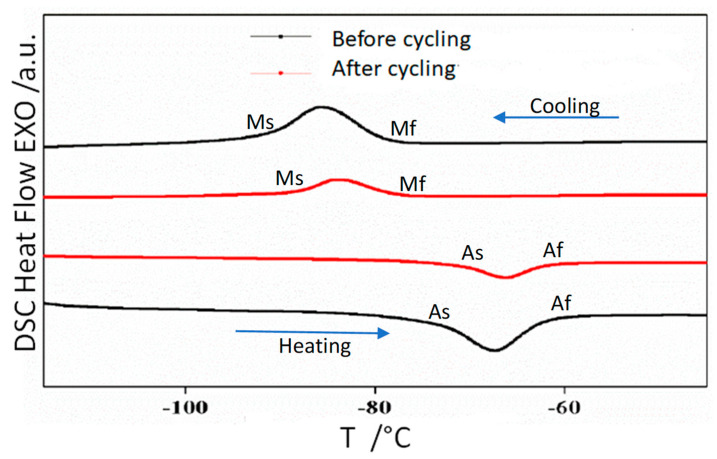
DSC reversible scans of alloy Ni_50_ Mn_36_ Sn_14_ before and after 100 thermal cycles.

**Figure 3:**
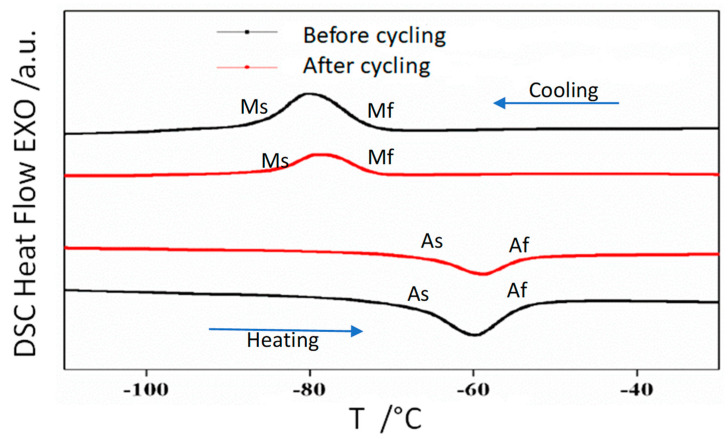
DSC reversible scans of alloy Ni_49_ Mn_36_ Sn_14_ Cu_1_ before and after 100 thermal cycles.

**Figure 4:**
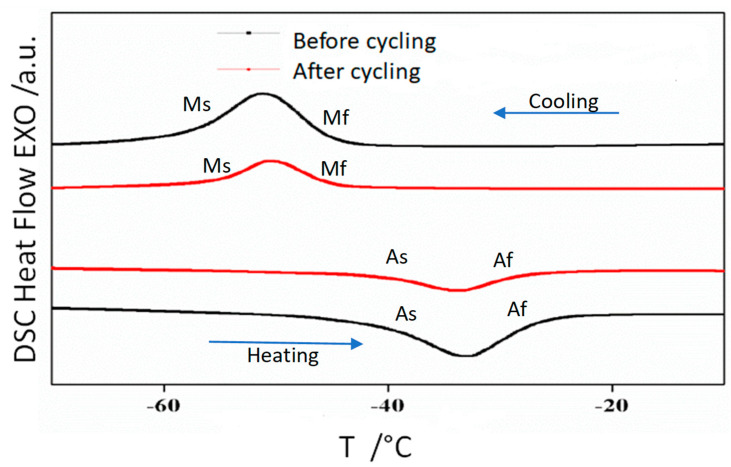
DSC reversible scans of alloy Ni_48_ Mn_36_ Sn_14_ Cu_2_ before and after 100 thermal cycles.

As the crystallographic phase is cubic at room temperature, in all samples only the austenite phase is detected. In order to determine the temperatures of the reversible austenite to martensite structural transformation continuous cooling-heating was performed. Figure 2, Figure 3 and Figure 4 show the DSC scans before and after thermal cycling of alloys Ni_50−*x*_ Mn_36_ Sn_14_ Cu_x_ (*x* = 0, 1, and 2), respectively.

The scans show that the addition of a minor amount of Cu shifts the transition temperatures to higher values. Thus, by controlling composition it is possible to design the production of alloys with transformation temperatures close to room temperature. A characteristic temperature is the equilibrium Gibbs temperature of the martensite structural transformation, T_o_, that is calculated from DSC scan analysis following the procedure described in reference [21]. The T_o_ values obtained before and after thermal cycling are provided in Table 2. Thermal cycling slightly increases the transformation temperatures.

The enthalpy and entropy values of the structural transformation (ΔH and ΔS respectively) are also given in Table 1. The enthalpy decreases with thermal cycling, the same effect is found in the entropy. These trends need to be confirmed for long term cycles and should be taken into account in the design of magnetocaloric devices.

### 3.3. Magnetic Analysis

**Figure 5:**
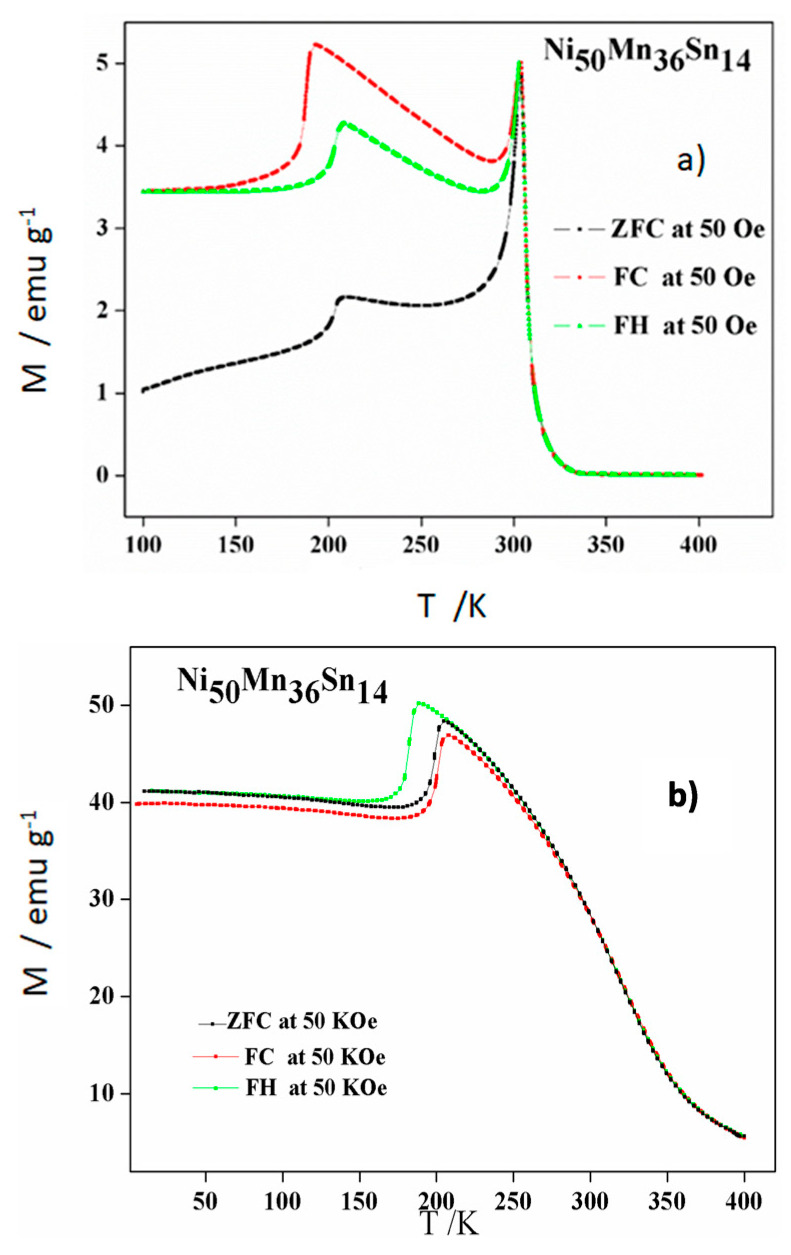
ZFC, FH, and FC thermomagnetic measurements of alloy Ni_50_Mn_36_Sn_14_. (**a**) 50 Oe and (**b**) 50 KOe.

**Figure 6:**
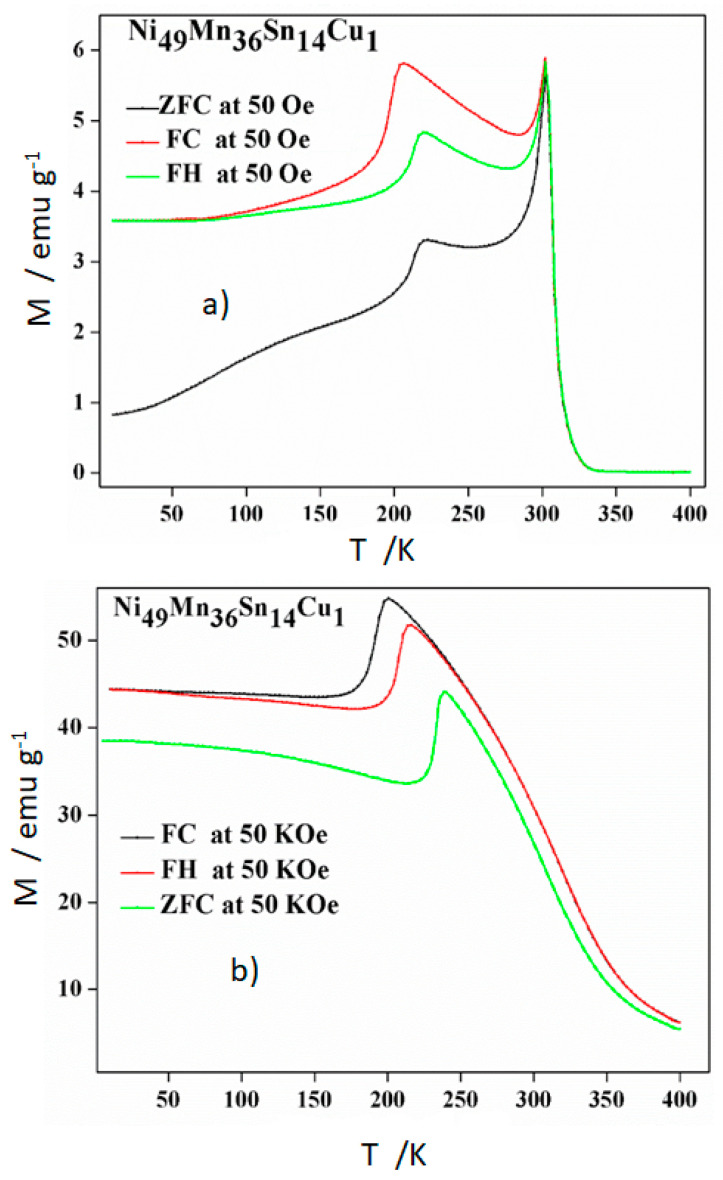
ZFC, FH and FC thermomagnetic measurements of alloy Ni_49_Mn_36_Sn_14_Cu_1_. (**a**) 50 Oe and (**b**) 50 KOe.

**Figure 7:**
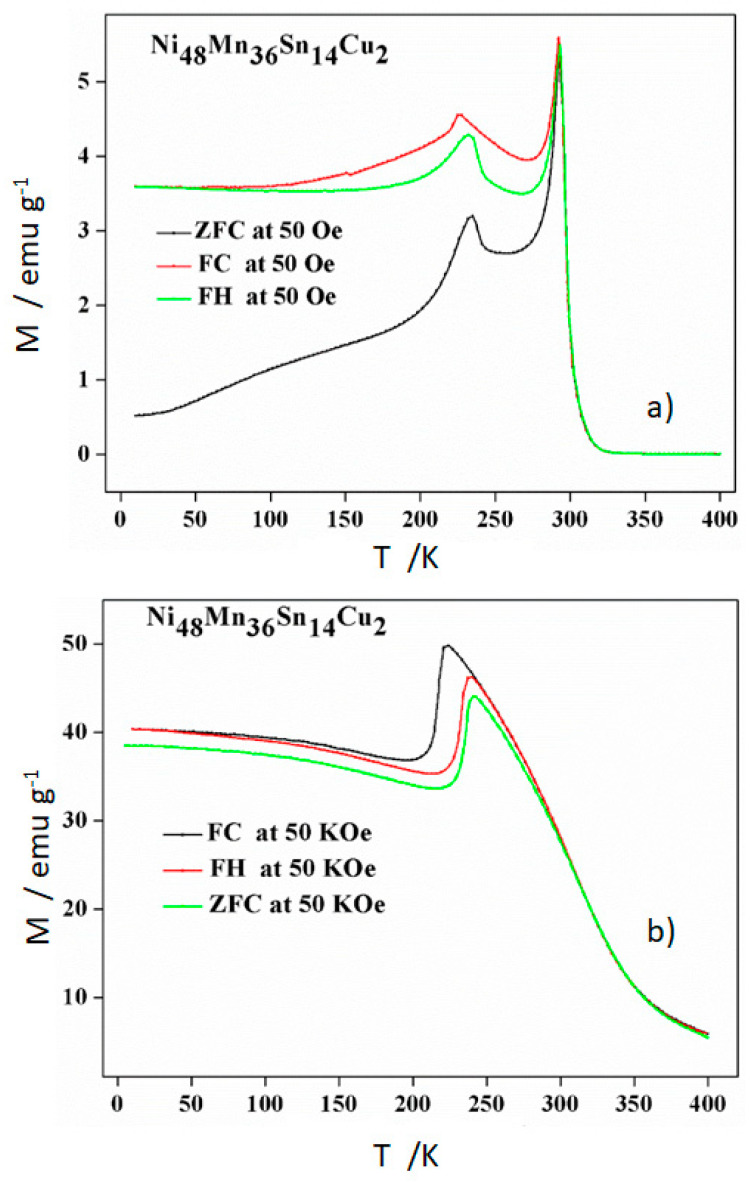
ZFC, FH, and FC thermomagnetic measurements of alloy Ni_48_Mn_36_Sn_14_Cu_2_. (**a**) 50 Oe and (**b**) 50 KOe.

Further, we will consider the magnetic properties of the alloys. Zero-field-cooling (ZFC), field-heating (FH), and field-cooling (FC) experiments have been performed at two different external magnetic fields to check the magnetic response at low (50 Oe) and high (50 KOe) magnetic fields. The thermomagnetic curves are represented in Figure 5, Figure 6 and Figure 7 for thermally cycled alloys Ni_50−*x*_ Mn_36_ Sn_14_ Cu_x_ (*x* = 0, 1, and 2), respectively.

As expected, through the process of the structural transition at 50 Oe, both FC and ZFC curves exhibit an irreversible behavior due to the obvious hysteresis between FC and ZFC curves, leading to the direct and inverse martensite transformation. It is known that this transformation requires overheating (martensite to austenite) and undercooling (austenite to martensite). This behavior was presented in such a thermal hysteresis between the FH and FC curves. Furthermore, the separation between the ZFC and FC scans below this ferromagnetic transition usually caused by spin glass state and/or the coexistence of antiferromagnetic and ferromagnetic states [22,23].

Regarding the influence of the Cu addition, it is clear that the Cu favors the increase of the temperatures of the structural transformation, confirming DSC data, whereas the Curie temperature remains early constant at around 310 K. Thus, this temperature was slightly increased (3–5 K) after thermal cycling [20]. The first effect was also detected in the samples before thermal cycling, whereas a slight decrease (up to 13 K) was found in the Curie temperature. From the results after cycling, we can remark that Cu addition could be a way to shift the structural transformation close to the magnetic transformation in order to favor the interplay between both transformations at low magnetic fields. It is well known that the total entropy change, ΔS, linked to the martensite transformation represents a limit to the attainable magnetic entropy change, and it has been phenomenologically established that it decreases exponentially with the increment of the generalized order parameter defined as the temperature interval between the Curie temperature of the austenite and the Gibbs equilibrium temperature of the structural transformation, T_C_ − T_o_ [10]. From such relation, it can be deduced that the closest the Curie temperature of the austenite to the equilibrium temperature, the largest the magnetic entropy change values can be achieved. Likewise, at 50 Oe the magnetization has a minor growth as Cu content increases. This tendency is not followed at 50 kOe, 1 at.% Cu addition increases magnetization, but 2 at.% Cu addition does not.

Furthermore, one can note that the martensite is more stable when increasing the applied magnetic field because in the alloy without Cu, it takes more energy to transform the austenite into martensite. The important shift in the temperatures of the structural transformation by the Cu addition detected in DSC scans is reduced in the thermomagnetic scans at 50 kOe. Thus, for the possible applications of these alloys it is important to analyse their magnetic response at the expected working magnetic field.

It is known that the increase of the magnetization shift between the martensitic and the austenite phases benefits the field induced phase transformation, especially close to temperatures close the austenite start formation [7]. In our study, an increase of the magnetization change was found as increasing Cu content, it is a signal that the energy difference between the austenite L2_1_ structure and the martensite structure increases by Cu doping [24]. Likewise, the calculated values of the temperature change due to an applied magnetic field of 1 T were 1.10, 1.30 and 1.16 K/T for the alloys with x = 0, 1, and 2 at.% of Cu, respectively. These values are slightly different than the calculated for the same specimens before thermal cycling: 1.28, 1.29, and 1.11 K/T. As there are close compositions, it is expected that the martensite phase was modulated 10M, as previously found in reference [20].

It can be concluded that the best alloy for applications linked to the magnetic shape memory and magnetocaloric effects is Ni_49_Mn_36_Sn_14_Cu_1_, as the elastocaloric behavior [25]. The non-continuous variation (as a function of Cu content) may be caused by hybridization or the non-entirely ferromagnetic band splitting [24].

**Figure 8:**
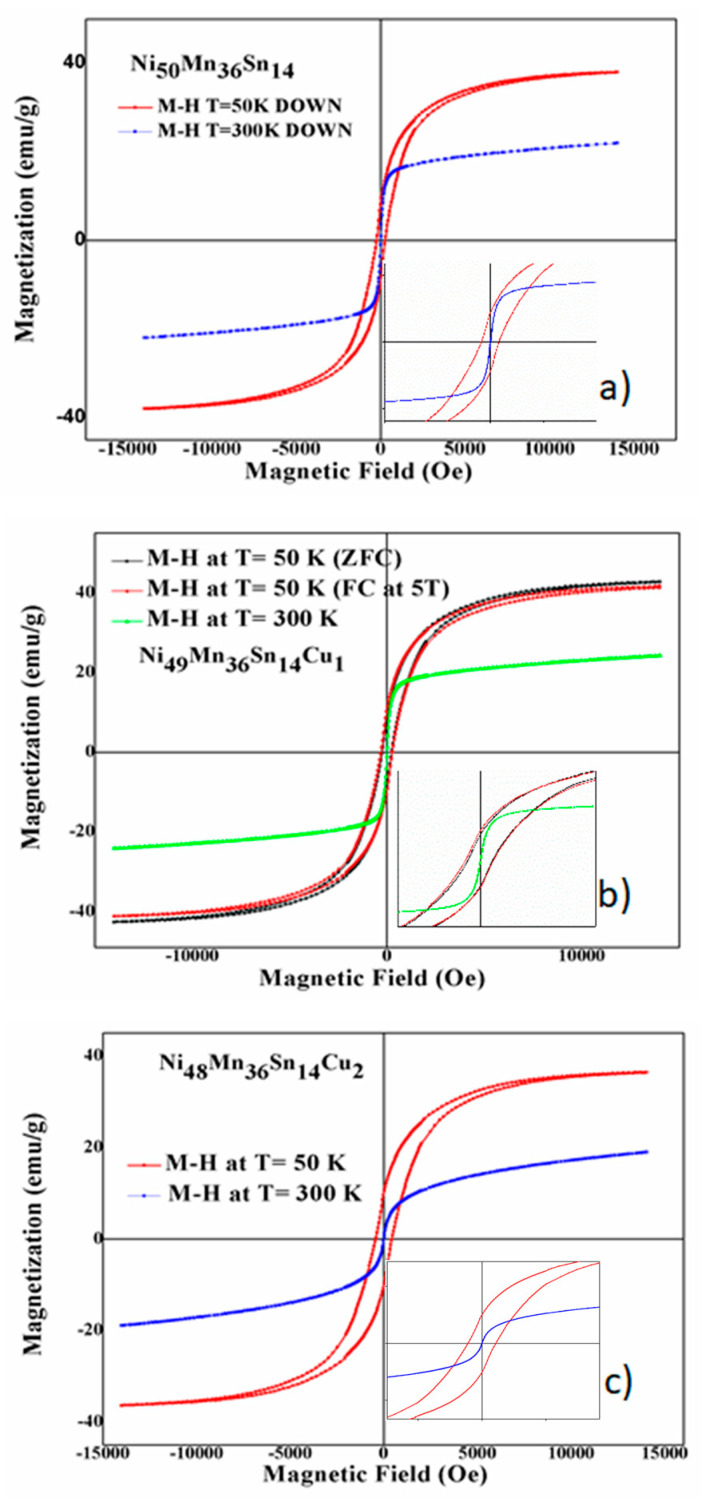
Magnetic hysteresis loops at room temperature of alloys: (**a**) Ni_50_Mn_36_Sn_14_, (**b**) Ni_49_Mn_36_Sn_14_Cu_a_ and (**c**) Ni_48_Mn_36_ Sn_14_Cu_2_.

Complementary magnetic analysis has been performed by performing magnetic hysteresis loops (50 K and 300 K). The results are shown in the Figure 8.

All the hysteresis loops at 50 K are clearly ferromagnetic. The measurements at 300 K show the decrease of the magnetization of saturation and the coercivity. The Cu addition provokes an increase in the coercivity and minor changes in the magnetization of saturation. The results are given in Table 3 and are similar to those obtained before thermal cycling. The increase on the coercivity with the increase of the Cu content can be provoked by the higher microstrain (Table 1 data).

## 4. Conclusions

Three alloys with nominal composition (at.%) Ni_50−*x*_Mn_36_Sn_14_Cu_x_ (*x* = 0, 1, and 2) were produced by melt-pinning as ribbon flakes. At room temperature all the samples have the austenite L2_1_ cubic Heusler structure. The L2_1_ order parameter decreases after cycling to values ranged between 73–75%. Thermal analysis detects the reversible structural transformation, Cu addition shifts this transformation to higher temperature. After thermal cycling (100 cycles) it was found a slight increase of the Gibbs equilibrium temperature of the structural transformation, this shift is reduced in the thermomagnetic scans at 50 kOe. Whereas the Curie temperature remains close to 310 K. The calculated magnetization/entropy change ratios were 1.10, 1.30 and 1.16 K/T for the alloys with x = 0, 1, and 2 at.% of Cu, respectively. Results show the interest to check the thermal stability and the magnetic response of these alloys due to the cycling working conditions linked to some of their specific applications and also the interest of Cu addition to shift the transition temperatures.

## Figures and Tables

**Table 1:** Crystallographic parameters obtained from XRD analysis of specimens before and after thermal cycling.

Alloy	Lattice Parameter/nm	Microstrain/%	Crystallite Size/nm
Ni_50_ Mn_36_ Sn_14_ (before)	a = 0.5983	0.650	114
Ni_50_ Mn_36_ Sn_14_ (after)	a = 0.5981	0.583	127
Ni_49_ Mn_36_ Sn_14_ Cu_1_ (before)	a = 0.5980	0.643	103
Ni_49_ Mn_36_ Sn_14_ Cu_1_ (after)	a = 0.5987	0.536	125
Ni_48_ Mn_36_ Sn_14_ Cu_2_ (before)	a = 0.5982	0.928	82
Ni_48_ Mn_36_ Sn_14_ Cu_2_ (after)	a = 0.5992	0.853	94

**Table 2:** Equilibrium Gibbs temperature, T_o_, and thermodynamic parameters (enthalpy ΔH, entropy, ΔS) of the structural transformation before and after thermal cycling (before values are calculated following the procedure and data provided in reference [21]). T_o_ is one half of M_s_ + A_f_.

Alloy	T_o_/K	ΔH/J g^−1^	ΔS/J g^−1^ K^−1^
Ni_50_ Mn_36_ Sn_14_ (before)	203.2	1.54	0.0077
Ni_50_ Mn_36_ Sn_14_ (after)	204.4	1.44	0.0073
Ni_49_ Mn_36_ Sn_14_ Cu_1_ (before)	211.5	1.61	0.0079
Ni_49_ Mn_36_ Sn_14_ Cu_1_ (after)	212.4	1.60	0.0077
Ni_48_ Mn_36_ Sn_14_ Cu_2_ (before)	237.0	2.53	0.0100
Ni_48_ Mn_36_ Sn_14_ Cu_2_ (after)	238.2	2.27	0.0096

**Table 3:** Magnetic parameters derived from Figure 8 hysteresis loops.

Alloy	Saturation Magnetization (emu/g) at 50 K	Saturation Magnetization (emu/g) at 300 K	Coercivity (Oe) at 50 K	Coercivity (Oe) at 300 K
Ni_50_ Mn_36_ Sn_14_	38.5	19.5	266	10.2
Ni_49_Mn_36_Sn_14_Cu_1_	40.0	21.5	275	10.0
Ni_48_Mn_36_Sn_14_Cu_2_	38.6	18.8	400	11.6

## Data Availability

Data can be requested from the authors.

## References

[1] Czaja P., Przewoznik J., Fitta M. (2021). Heat treatment effect on the evolution of magnetic properties of martensite in magnetic shape memory Ni_48_Mn_39.5_Sn_9.5_Al_3_ Heusler alloy ribbons. Mater. Res. Bull..

[2] Navarro-García J.D., Llamazares J.L.S., Camarillo-García J.P. (2021). Synthesis of highly dense spark plasma sintered magnetocaloric Ni-Mn-Sn alloys from melt spun ribbons. Mater. Lett..

[3] Chabri T., Ghosh K., Mukherjee D., Nath T.K. (2020). Role of interplay of austenite and martensite phase fractions on the magnetocaloric and magnetoresistance effects across the martensite transition in Ni_45_Mn_44_Sn_7_In_4_Heysler alloy near room temperature. J. Appl. Phys..

[4] Ling Y.C., Hu Y., Wang H.B., Niu B., Chen J.W., Liu R.B., Yuan Y., Wang G.Y., Wu D., Xu M.X. (2021). Strain control of phase transition and exchange bias in flexible Heusler alloy thin films. ACS Appl. Mater. Interface.

[5] El-Khatib S., Bhatti K.P., Srivastava V., James R.D., Leighton C. (2019). Nanoscale magnetic phase competition throughout the Ni_50-x_Co_x_Mn_40_Sn_10_ phase diagram: Insights from small angle neutron scattering. Phys. Rev. Mater..

[6] Shen Y., Wei Z.Y., Shen Q., Zhang Y.F., Liu J. (2019). Orientation dependent elastocaloric effect in directionally solidified Ni-Mn-Sn alloys. Scrip. Mat..

[7] Louidi S., Suñol J.J., Ipatov M., Hernando B. (2018). Effect of Co doping on martensitic transformations and the magnetic properties of Ni_50-x_Co_x_Mn_37_Sn_1 3_ (x = 1, 2, 3) Heusler ribbons. J. Alloys Comp..

[8] Khan M., Jung J., Stoyko S.S., Mar A., Quetz A., Samanta T., Dubenko I., Ali N., Stadler S., Chow K.H. (2012). The role of Ni-Mn hybridization on the martensitic phase transitions in Mn-rich Heusler alloys. App. Phys. Lett..

[9] Bachaga T., Zhang J., Khitouni M., Suñol J.J. (2019). NiMn-based Heusler magnetic shape memory alloys: A review. Inter. J. Adv. Manuf. Tech..

[10] Deltell A., El-Moez A.A.M., Álvarez-Alonso P., Ipatov M., Andrés J.P., González J.A., Sánchez T., Zhukov A., Escoda M.L., Suñol J.J. (2021). Martensitic transformation, magnetic and magnetocaloric properties of NiMnFeSn Heusler ribbons. J. Mater. Res. Tech..

[11] Unzueta I., López-García J., Sánchez-Alarcos V., Recarte V., Pérez-Landazabal J.I., Rodríguez-Velamazán J.A., Garitaonandia J.S., Garcia J.A., Plazaola F. (2021). Testing the applicability of Sn 119 Mössbauer spectroscopy for the internal stress study in ternary and Co doped Ni-Mn-Sn metamagnetic alloys. Metals.

[12] Wang L.B., Xuan H.C., Liu S.L., Cao T., Xie Z.G., Liang X.H., Chen F.H., Zhang K.W., Feng L., Han P.D. (2020). Enhanced elastocaloric effect and mechanical properties of Gd-doped Ni–Mn–Sn-Gd ferromagnetic shape memory alloys. J. Alloys Comp..

[13] Dadda K., Alleg S., Suñol J.J., Bessais L., Hlil E.K. (2020). Structure, magnetocaloric effect and critical behavior in Ni_50_Mn_30_(Sn,In)_20_ Heusler alloys. J. Super. Nov. Magn..

[14] Varzaneh A.G., Kameli P., Sarsari I.A., Zavareh M.G., Mejia C.S., Amiri T., Skorski Y., Luo J.L., Etsell T.H., Chernenko V.A. (2020). Magnetic and magnetocaloric properties of Ni_47_Mn_40_Sn_13-x_Zn_x_ alloys: Direct measurements and first-principles calculations. Phys. Rev. B.

[15] Villa E., Maziarz W., Wojcik A., Nespoli A., Lazpita P., Hosoda H., Chernenko V. (2020). Martensitic transformation and structural states in Ni_44.0_Mn_43.5_Sn_12.5−x_Al_x_ (x = 1, 2, 3 at.%) magnetic shape memory alloys prepared by vacuum hot pressing. J. Alloys Comp..

[16] Wederni A., Ipatov M., Pineda L., Escoda L., González J.M., Khitouni M., Suñol J.J. (2020). Martensitic transformation, thermal analysis and magnetocaloric properties of Ni-Mn-Sn-Pd alloys. Processes.

[17] Kirat G., Aksan M.A. (2021). Investigation of martensitic transformation and magnetoresistance properties of Cu substituted Ni-Mn-Sn-B melt spun ribbons. J. Magn. Magn. Mater..

[18] Han B.L., Tan C.L., Zhao L., Zhao W.B., Ma T.Y., Wang C., Zhang K., Tian X.H. (2021). Investigation on phase structure and magnetic properties of high-temperature Ni-Pt-Co-Mn-Sn magnetic shape memory alloys by first-principles calculations. Comp. Mater. Sci..

[19] Qu Y., Gràcia-Condal A., Mañosa L., Planes A., Cong D., Nie Z., Ren Y., Wang Y. (2019). Outstanding caloric performances for energy-efficient multicaloric cooling in a Ni-Mn based multifunctional alloy. Acta Mater..

[20] Wederni A., Ipatov M., Pineda E., Suñol J.J., Escoda L., González J.M., Alleg S., Khitouni M., Zuberek R., Chumak O. (2020). Magnetic properties, martensitic and magnetostructural transformations of ferromagnetic Ni-Mn-Sn-Cu shape memory alloys. Appl. Phys. A.

[21] Coll R., Saurina J., Escoda L., Suñol J.J. (2018). Thermal analysis of Mn_50_Ni_50-x_(Sn,In)_x_ Heusler shape memory alloys. J. Therm. Anal. Calorim..

[22] Wang H., Zhang H., Tan Y.W., Huo D. (2021). Spin glass feature and exchange bias effect in metallic Pt/antiferromagnetic LaMnO_3_ heterostructure. J. Phys. Condens. Matter..

[23] Zhang K., Tian X., Tan C., Guo E., Zhao W., Cai W. (2018). Designing a new Ni-Mn-Sn-Fe ferromagnetic shape memory alloy with excellent performance by Cu addition. Metals.

[24] Yuzuak E. (2021). The magnetothermal characterization of Ni-Cu-Mn-Sn alloy. Mater. Res. Bull..

[25] Castillo-Villa P.O., Mañosa L., Planes A., Boto-Parra D.E., Sánchez-Llamazares J.L., Flores-Zúñiga H., Frontera C. (2013). Elastocaloric and magnetocaloric effects in Ni-Mn-Sn(Cu) shape memory alloy. J. Appl. Phys..

